# Proliferative vitreoretinopathy: an update on the current and emerging treatment options

**DOI:** 10.1007/s00417-023-06264-1

**Published:** 2023-10-16

**Authors:** Lorenzo Ferro Desideri, Dmitri Artemiev, Souska Zandi, Martin S Zinkernagel, Rodrigo Anguita

**Affiliations:** 1 Department of Ophthalmology, Inselspital, Bern University Hospital, University of Bern, Freiburgstrasse 15, CH-3010 Bern, Switzerland; 2 Department for BioMedical Research, University of Bern, Murtenstrasse 24, CH-3008 Bern, Switzerland; 3 Bern Photographic Reading Center, Inselspital, Bern University Hospital, University of Bern, Bern, Switzerland; 4 Moorfields Eye Hospital NHS Foundation Trust, London, UK

**Keywords:** PVR, Proliferative vitreoretinopathy, Vitrectomy, Steroids, Methotrexate

## Abstract

Proliferative vitreoretinopathy (PVR) remains the main cause of failure in retinal detachment (RD) surgery and a demanding challenge for vitreoretinal surgeons. Despite the large improvements in surgical techniques and a better understanding of PVR pathogenesis in the last years, satisfactory anatomical and visual outcomes have not been provided yet. For this reason, several different adjunctive pharmacological agents have been investigated in combination with surgery. In this review, we analyze the current and emerging adjunctive treatment options for the management of PVR and we discuss their possible clinical application and beneficial role in this subgroup of patients.
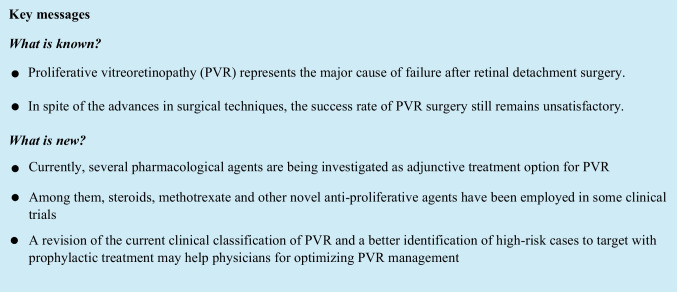

## Introduction

Proliferative vitreoretinopathy (PVR) is the main cause of failure after retinal detachment (RD) surgical repair [1]. PVR is caused by the formation and contraction of proliferative cellular membranes in the vitreous cavity on both retinal sides, leading to the possibility of a tractional RD with fixed retinal folds [2]. The Retina Society Terminology Committee first introduced the definition of PVR, suggesting a staging classification (A, B, C, D) in relation to disease severity. In detail, PVR stage A is minimal; B is moderate with surface retinal wrinkling, rolled edges of the retinal, retinal stiffness, and vessel tortuosity; C is marked with full-thickness retinal folds (C1 one quadrant, C2 two quadrants, C3 three quadrants); and D is a massive PVR with fixed retinal folds in four quadrants (D1 wide funnels shape, D2 a narrow funnel shape, D3 closed funnel without view of the optic disc) [3]; however, several limitations were still present with this definition, and further modifications were subsequently proposed, including one in 1989 by the Silicone Study Group, describing the location (anterior or posterior) and the type of contraction; this was followed by a further classification in 1991, in which a more detailed description of the anterior and posterior PVR was provided [3, 4]. In the last years, despite the improvements in vitreoretinal surgery techniques, the incidence of PVR has remained stable, ranging from 5 to 10% of the cases after RD repair surgery. It has been reported that almost 77% of cases of PVR occurs within 1 month after RD surgery and 95% of them within 3 months [5]. Table 1

**Table 1:** Level of evidence and grade of recommendations for adjunctive drugs for treating proliferative vitreoretinopathy according to the Oxford Centre for Evidence-Based Medicine (OCEBM)

investigated treatment	Level of evidence	Grade of recommendations
Steroids	1b	C
MTX	2b	C
Daunomycin	1b	D
5-FU/LMWH	2b	D
RA	2b	D
MMC	3b	C
Anti-VEGF agents	1a	D

*5-FU/LMWH* = 5-fluorouracil and low-molecular weight heparin; *MMC* = mitomycin; *MTX* = methotrexate; *RA* = retinoic acid

Grade of Recommendation (A–D)

Level of evidence (1a–4)

https://www.cebm.ox.ac.uk/resources/levels-of-evidence/oxford-centre-for-evidence-based-medicine-levels-of-evidence-march-2009

Surgical repair currently represents the mainstay for the treatment of PVR [6]. Although there have been recent advances in the surgical management of RD with PVR, the recurrence rate of RD due to PVR itself continues to be a significant limitation to success [7]. In this regard, the Silicone Oil Study showed a limited success rate in PVR surgery (35–42%) and unsatisfactory visual outcomes [8]. In series with established PVR at presentation, the primary overall success rate has been reported between 43 and 69%; although cases can be treated with good anatomical results, visual outcomes are often less favorable, and more reoperations are required compared with standard retinal detachments [9].

Previous studies have referred a best-corrected visual acuity (BCVA) of ≥ 5/200 in 40–80% of the patients following to RD repair with PVR [10]. Previous clinical studies showed that eyes that developed PVR resulting in worse visual outcomes [11, 12]. Poorer visual outcomes have been linked to some clinical features, including the presence of anterior PVR and a history of multiple surgeries.

These risk factors may cause pathological microscopical alterations in macular region, including the rearrangement of the retinal pigment epithelium (RPE), macular edema, secondary epiretinal membranes, and subretinal fibrosis [13].

To improve the still unsatisfactory anatomical and functional outcomes of PVR surgery, several novel additional agents are being studied. The aim of this review is to give an overview of the past and current investigational treatment options and discuss their possible clinical application for the management of PVR.

## Methodology

A literature search was conducted to find all the published studies examining these topics from inception until April 2023. The following digital databases were adopted: Medline, PubMed, Science Citation Index via Web of Science, and the Cochrane Library. The following search terms were used: “rhegmatogenous retinal detachment,” “proliferative vitreoretinopathy,” “PVR” alone or in combination with “treatment,” “therapy,” “drugs,” “methotrexate,” “corticosteroids,” “anti-VEGF,” “anti-proliferative agents,” “anti-neoplastic agents,” “daunorubicin.” Current research registers (such as http://www.cliniclatrials.gov) were also examined in the research. “Essential” terms AND OR were used yielding a total of 121 articles. Only articles in English language were considered. The remaining abstracts and articles were reviewed by the authors and were included based on their relevance to this review article. In addition, the primary references mentioned in the papers were also reviewed.

## Pathogenesis

The pathogenesis of PVR is complex and relies on numerous interactions that are not yet fully understood. Various cell types are involved, such as glial cells, retinal pigment epithelial cells (RPE), inflammatory cells, and fibroblasts, which are regulated by numerous cytokines, inflammatory factors, and growth factors [14–16].

Cell death pathways, including apoptosis and programmed necrosis, have also been found to be involved in retinal photoreceptor degeneration and cell death. These processes may play an important role in the development of proliferative vitreoretinopathy and provide clues to the imbalance in fibrosis formation during wound healing [15].

In recent years, it has been found that the influence of Müller glia on other retinal cells and their involvement in the biochemical cascade during PVR are more important than previously thought. The response of the Müller glia during PVR resembles the response to a pathogenic stimulus, resulting in hypertrophy, increased proliferation, and upregulation of intermediate filaments nestin, vimentin, and glial fibrillary acidic protein (GFAP) [17].

Müller glia cells respond to exogenous factors and release a variety of signaling molecules, which contribute to the regulation of retinal tissue inflammation and local immunity, including pro-inflammatory cytokines such as interleukin 8 (IL-8), interleukin 6 (IL-6), tumor necrosis factor α (TNF-α), and chemotactic molecules monocyte chemoattractant protein-1 (MCP-1) and macrophage inflammatory protein-1 alpha (MIP-1α) [18]. Moreover, an increased expression of toll-like receptors and upregulation of major histocompatibility complex class II (MHC II) molecules in the context of retinal detachment and oxidative stress has been demonstrated [19]. Among the pro-inflammatory cytokines, CXCL5 has been reported to be a potent biomarker for the de novo development of postoperative PVR [20]. This background supports the assumption that humoral and cellular responses play an important role in the pathogenesis of PVR [21, 22].

Furthermore, it is known that RPE cells make a major contribution to the development of PVR [21]. In ultrastructural studies, they have been shown to represent 50–90% of all cells obtained from examination of the subretinal membranes from enucleated eye specimens [16]. RPE cells can release the immunologically similar proteins such as platelet-derived growth factor (PDGF), fibroblast growth factors (FGFs), and transforming growth factor beta (TGF-β), which are involved in the autocrine regulation of growth control [23] In addition, it has been postulated that a portion of the fibroblast cells may derive from the RPE cells, which undergo transdifferentiation from an epithelial to a mesenchymal phenotype; this biological process allows RPE cells to acquire new different functions, including the capability to migrate and invade other tissues, resistance to apoptosis, and the possibility to produce metalloproteases and remodel the extracellular matrix [24].

Other preclinical studies have highlighted that macrophages are important players in the pathogenesis of PVR. Their presence in the vitreous has been related to a higher risk to develop PVR [25]. In fact, these cells are more frequent in the vitreous body of those patients developing PVR after surgery in comparison with those who will not develop PVR [26]. Moreover, macrophages not only display a pro-inflammatory activity, but they can also mediate the apoptosis of photoreceptor cells through MCP-1 [27].

The complex interplay between different cell types (RPE cells glial cells and macrophages), cytokines, and other mediators involved in the pathogenesis of PVR creates a feedback loop maintaining the disease process. This complicated network of cellular interactions and signaling pathways makes the development of effective therapeutic interventions for PVR a challenging task.

## Adjunctive treatment options

Nowadays, no pharmaceutical agents have been approved as adjunctive treatment in combination with surgery for treating PVR. Considering the pathogenetic mechanism of the disease, several different drugs targeting inflammation, cell proliferation, and fibrosis processes have been examined. Among others, corticosteroids, methotrexate, and other different anti-proliferative agents have shown promising results in preclinical models, and their application in clinical practice is currently under investigation [7].

### Steroids

The first agents studied for the management of PVR were corticosteroids, considering their broad anti-inflammatory and anti-proliferative activity, the multiple modalities of administration, and the lack of evidence in terms of retinal toxicity [28]. Animal models have provided evidence on the efficacy of steroids in preventing and/or treating PVR. Chandler et *al.* carried out an experimental study in rabbit eyes. They refined a rabbit model of PVR in which the vitreous was compressed and partially detached from the retinal surface and small amounts of tissue-cultured homologous fibroblasts (25,000) were scattered over the vascularized part of the retina. In this model, it was documented that triamcinolone acetonide at 2 mg was effective in reducing the incidence of PVR-related RD from 90 to 56%. This effect was less pronounced than in previous models with intact vitreous. Moreover, higher doses of the corticosteroid had no additional effect on the decrease of RD, indicating an optimal dosage of 2 mg [29].

In another similar preclinical study on rabbits, the incidence of RD was examined with tissue-cultured fibroblast resembling a PVR model. It was found that the simultaneous intravitreal injection of triamcinolone acetonide with the cells lead to a reduction of RD from 93 to 75% after 28 days. As a prophylactic agent, when the steroid was administered 24 h before experimentally inducing PVR, RD decreased from 85% in the control group to 43% in the treated one [30].

In spite of the promising outcomes documented from preclinical models, clinical studies on corticosteroids for the management of PVR have provided contradictory results.

A randomized, clinical trial examined the clinical efficacy of adjunctive triamcinolone on 75 eyes with RD and PVR grade C undergoing vitrectomy in combination with silicone oil tamponade. They found that there were no significant differences between patients receiving the intraoperative steroid in comparison with controls [31].

Munir et *al.* analyzed the clinical efficacy of intravitreal triamcinolone in conjunction with vitrectomy and silicone oil injection for treating complicated PVR. Thirteen eyes of 12 consecutive patients were enrolled. The study subjects received 0.1 mL intravitreal injection of triamcinolone acetonide at the dose of 4 mg. Contradictory results in terms of visual benefits were reported (BCVA improved in 4 eyes, remained stable in 5 eyes, and decreased in 4 eyes). Retina was attached in 10 out of 13 eyes after almost 5 months of follow-up, and 8 out of 13 eyes did not any clinical signs of reproliferation [32].

Koerner et *al.* reported 24 with diagnosis of RD and advanced PVR grade C2. They underwent pars plana vitrectomy and oil tamponade. A visual improvement in the whole study cohort and a successfully reattached retina in 87.5% of them at the end of the follow-up were reported [33].

The administration of steroids in PVR has been studied also via systemic intake; however, in a prospective study, oral prednisolone showed a weaker clinical effect in reducing fibrosis in comparison with that known from experimental conditions with intravitreal triamcinolone acetonide and in improving visual outcomes in patients with PVR [34].

Sustained delivery systems have been shown to maintain an active vitreous concentration of the drug for a longer time period, referring promising results in preclinical studies [35, 36]. In a 2-year, randomized, prospective study, 140 patients underwent vitrectomy surgery with silicone oil for RD with PVR grade C, and they were assigned to either intraoperative dexamethasone 0.7 mg implant or placebo. Anatomic success was similar for both groups (49.3% vs 46.3% in the dexamethasone and control group, respectively, *p*=0.733), and after 6 months BCVA was 38.3 and 40.2 ETDRS letters in the dexamethasone and control groups, respectively. Hence, in this study, sustained-release dexamethasone implant did not show any significant anatomical and functional results for treating PVR [37].

PVR is a frequent complication in open globe trauma (OGT); for this reason, triamcinolone acetonide has been studied in patients undergoing vitrectomy for OGT and has shown promising results in terms of visual results when administered as an adjunctive agent [38]. However, A phase 3, multicenter, double-masked randomized controlled trial of patients undergoing vitrectomy following OGT comparing adjunctive TA (intravitreal and subtenons) against standard care failed to show any treatment benefit [39].

In a small, randomized, clinical trial, triamcinolone was combined with heparin administered with an infusion during the vitrectomy; however, the combination therapy failed to provide clinical benefits in established PVR [40].

### Methotrexate

Methotrexate (MTX) is a folate antagonist, which exerts an anti-proliferative activity, by reducing DNA replication and cell proliferation, and an anti-inflammatory activity, by stimulating adenosine release [41]. *In vitro* studies on PVR membrane isolated from human patients have revealed that MTX could decrease RPE cells proliferation and migration, inducing also cell apoptosis; moreover, differently from 5-fluoruracil, MTX was not related to photoreceptor cells toxicity [42, 43].

In clinical studies, MTX has been examined with a different dosage regimens (ranging from 100 to 400 μg) in different delivery modalities, including single and multiple postoperative intravitreal injections and intraoperative infusions [44].

Early clinical studies reported encouraging results with intraoperative single injection MTX in PVR grade C during vitrectomy with silicone oil; in a case series on 10 patients, a success rate of 80% was documented after an average 25-month follow-up period and an increase of BCVA from hand motion to 20/200 on median; only one case of superficial punctate keratopathy (SPK) was documented [45]. Similar results were reported in another small retrospective study [46]. A prospective case series examined 11 patients undergoing vitrectomy with oil tamponade and multiple MTX postoperative injections for RD with PVR grade C at presentation with or without a previous history for RD. They showed that the retina remained attached in the whole cohort after 9 months (only 2 relapsing cases without posterior pole involvement) and a slight median gain in BCVA. No systemic or local AEs were reported [47].

Falavarjani et *al.* analyzed the role of intrasilicone oil injection of MTX at 250 μg, given the end of vitrectomy surgery for RD with PVR grade C at presentation in a sample of 44 patients. The incidence of PVR-related retinal redetachment was measured. They found that the redetachment rate associated with PVR was lower in the MTX group in comparison with controls; however, the difference was not statistically significant. BCVA did not differ between the 2 groups as well [48].

A previous study provided evidence on the clinical efficacy of intravitreal MTX infusion at 400 μg in eyes with high risk for PVR development, showing a reduction in PVR incidence following the infusion performed during the vitrectomy for RD. In fact, 26 of 29 MTX-treated eyes (90%) remained attached, while the remaining 3 needed another reattachment surgery. Three additional eyes (10%) developed recurrent limited PVR without recurrent RD. This drug delivery modality was also well-tolerated by the study subjects [49].

In an interventional case series, the efficacy of MTX intraoperative infusion at 400 μg in 30 patients with PVR was analyzed. Average BCVA increased from 20/447 to 20/204 after 4 months. Eighty percent of the patients had an attached retina after the first surgery and a success rate of 93.3% was referred after the second surgery [50].

In another comparative study, consecutive patients who had vitrectomy for RD were included and divided into 3 groups, including groups I (established PVR), II (high risk of PVR), and III (no risk of PVR), and compared to controls. The intravitreal MTX infusion at 400 μg confirmed the prophylactic activity of this drug when administered during the vitrectomy, reporting superior visual results in patients at high risk of developing PVR in comparison with controls (vitrectomy alone); however, when PVR was already established, the infusion of MTX did not provide any clinical benefit, including the increase of the retinal reattachment rate [51].

The most common adverse event (AE) associated with the use of intravitreal MTX is the development of a corneal epitheliopathy with a reported incidence of around 15% of the patients. This incidence is decreased when a longer interval between MTX injections is applied [44].

Currently, preliminary results of the randomized, multicenter, phase III GUARD trial (NCT04136366) have been revealed. In this study, patients were randomized into 2 different groups: one treated with MTX 0.8% given 13 times every 16 weeks after having completed the vitrectomy or the other one underwent vitrectomy alone without MTX. Inclusion criteria were the presence of patients undergoing vitrectomy due to PVR or open globe injury. Preliminary results showed that the MTX 0.8%–treated group had clinical benefits over routine surgical care in RD rate, risk of developing hypotony, complete retinal attachment, and epiretinal membrane formation (*p*=0.047) after 6 months. No functional differences and safety concerns were found between the groups [52].

### Other anti-proliferative and anti-neoplastic agents

Daunomycin is an anthracycline exerting an inhibitory activity on cell proliferation and migration, which has shown to be effective against PVR in animal models [53, 54].

The Daunomycin Study Group was a randomized, multicenter, prospective trial evaluating the clinical efficacy and safety of adjunctive daunomycin intraoperative infusion in comparison with vitrectomy surgery alone in 286 patients with PVR grade C2. After 6 months from surgery, complete retinal reattachment was achieved by 62.7% of the patients in the daunomycin as opposed to 54.1% in the control group (*p*=0.07). In the daunomycin group, a lower number of reoperations was needed within 1 year to have the same reattachment rate (80.2% in the study subjects vs 81.1% in controls, respectively, *p*=0.005). No differences in terms of BCVA were reported between the groups and daunomycin resulted well tolerated by patients [55]. No further clinical trials on daunomycin were performed, because of the promising but non-significant differences in terms of primary outcomes, the difficulty to obtain and utilize an anti-neoplastic agent in ophthalmologic setting, and the concerns on safety outcomes.

Animal studies have provided evidence on the role of low molecular weight heparin (LMWH) in reducing the rate of tractional RD; furthermore, a decrease in postoperative fibrin after vitrectomy has been documented [56, 57]. In addition, 5-fluorouracil (5-FU), another anti-neoplastic antimetabolite agent, has proven to decrease the rate of PVR in animal models [58]. Given this background, the combination therapy 5-FU and LMWH was investigated in the prevention of PVR in a randomized, multicenter trial. Both the 5-FU and LMWH medication group and the placebo group each had 87 patients. When compared to the placebo group, the incidence of postoperative PVR was considerably lower in the 5-FU and LMWH therapy group (*p* = 0.02). Postoperative PVR occurred in 26.4% (23/87) of the placebo group and 12.6% (11/87) of the 5-FU and LMWH group. In the 5-FU and LMWH group, there were 19.5% (17/87) patients who underwent multiple procedures, and there were 52.9% (9/17) reoperations brought on by PVR. In the placebo group, there were 25.3% (22/87) patients who underwent multiple procedures, and there were 72.7% (16/22) reoperations brought about by PVR. There was no statistically significant change in the visual acuity between the 2 groups. Evidence on the significant decrease in the incidence of postoperative PVR and in reoperation rate in patients receiving 5-FU and LMWH in comparison with controls was provided [59].

Recently, another randomized, double-blinded, multicenter trial assigned 325 patients to either 5-FU/LMWH combination therapy or placebo. Overall, no significant differences were found in the rate of PVR between adjuvant therapy with 5-FU and LMWH and placebo in patients with RD [60].

*In vitro* studies have also shown that retinoic acid inhibits the growth of RPE cells [61].

In this regard, a randomized, prospective study analyzed the clinical efficacy of 13-cis-retinoic acid given at the dose of 10 mg orally twice daily for 8 weeks postoperatively in patients with primary RD and PVR. Thirty-five patients were included in the study with 16 of them treated with retinoic acid and 19 in the placebo control group. They showed that retinal attachment was obtained in 93.8% of the patients in the retinoic acid group vs 63.2 % in controls (*p*=0.047). Patients in the treated arm had also a lower incidence of macular pucker formation (18.8% vs 78.9% in controls, *p*=0.001) and a greater rate of ambulatory vision (56.3% vs 10.5% in controls, *p*=0.009) [62]. The DELIVERY prospective, open-label trial examined the effect of low-dose, oral isotretinoin in decreasing the risk PVR after RD surgery. Two cohorts were included in the study: 51 eyes with recurrent PVR-related RD and 58 eyes with primary RRD associated with high-risk characteristics for developing PVR. Eyes in the study group were treated with 20 mg of oral isotretinoin once daily for 12 weeks starting the day after surgical repair. The single surgery anatomic success rate was 78.4% in isotretinoin treated eyes versus 70.0% in controls (*p*=0.358) in established PVR. In eyes with RD at high risk for developing PVR, the single surgery success rate was 84.5% versus 61.1% (*p*=0.005) for eyes treated with isotretinoin versus controls. The most common AE associated with isotretinoin was the onset of dry skin and mucus membranes in almost 98% of the patients; however, no severe AEs were documented in both arms [63]. Despite the promising results, no further large studies have been conducted investigating the role of retinoic acid/isotretinoin, probably due to safety concerns issues.

Some studies have shown that mitomycin C, an anti-proliferative agent, may have a beneficial effect in the reduction of post-traumatic PVR rate and in the improvement anatomical and functional results [64, 65]; however, further randomized, controlled studies are needed to provide more evidence in this regard.

Several other anti-proliferative molecules have shown promising results *in vivo* and in *in vitro* models, including taxol, colchicine, glucosamine, alkyphosphocoline, and palomide; however, no clinical trials have been conducted in order to assess their clinical efficacy and safety profile yet [1].

Currently, given the anti-fibrotic and anti-proliferative activity of topotecan, an ongoing prospective, phase II trial (NCT05523869) is investigating its clinical efficacy and safety profile administered intravitreally in patients with PVR-induced recurrent RD [66].

Another prospective, non-randomized, pilot study investigated the role of postoperative curcumin infusion in patients with PVR after RD. Fifteen patients received either curcumin-HSA infusion, prednisolone infusion, or no therapy. Among them, only one patient in the prednisolone group developed PVR, while the others did not. Curcumin infusion showed promise as a safe option for PVR risk reduction, but larger randomized trials are needed for further investigation [67].

### Anti-VEGF agents

Recent studies have highlighted the role of growth factors in the pathogenesis of PVR. In particular, the vascular endothelial growth factor (VEGF A) has been reported to activate the platelet-derived growth factor (PDGF) receptor α, which is involved in PVR pathogenesis [68]. Animal models have provided analyzed the role of ranibizumab, an anti-VEGF agent targeting all the isoforms of VEGF-A, for preventing the onset of PVR. Ranibizumab was shown to be effective in reducing the bioactivity of vitreous in experimental animals with PVR and protected rabbit from PVR development [69].

Despite the promising outcomes obtained in animal models, poorer clinical results have been shown in clinical studies. A prospective study examined the effect of serial intrasilicone oil bevacizumab injections (1.25 mg/0.05 mL) on BCVA and anatomical success rate in patients operated for PVR-induced RD. They revealed no significant difference in final BCVA, retinal reattachment rate, and epiretinal membrane formation between bevacizumab-treated patients and controls [70]. Similar outcomes were reported in a previous study [71]. In this regard, a meta-analysis analyzing 133 studies stated that intravitreal injection of bevacizumab in vitrectomy for patients with PVR-related RD was not effective in lowering retinal redetachment rate or improving visual acuity [72].

## Conclusions

PVR remains a challenging condition and the main cause of RD failure for vitreoretinal specialists. Although several different advantages in vitreoretinal surgery techniques have been obtained, to date, functional and anatomical outcomes in patients with PVR are still unsatisfactory [7].

Due to this unmet need, various adjunctive treatment options have been investigated for the management of PVR in preclinical models and some of them experimented in clinical studies.

Among them, steroids in various delivery systems, methotrexate, and other anti-proliferative agents have been employed in some clinical trials [1, 5]; however, due to the presence of contradictory results in terms of clinical efficacy and the small, non-randomized nature of the majority of these studies, no consistent evidence can be drown with the current data.

PVR is a multifactorial and complex disorder, whose pathogenesis is associated with the presence of several different cells (RPE cells glial cells and macrophages) and cytokines exerting pro-inflammatory and profibrotic activity [7]. A better understanding of the pathogenic process and the identification of key molecules involved in PVR onset may address us in the selection of the therapeutic targets, which should be focused on the retinal response.

Furthermore, to improve PVR clinical outcomes, a revision of the current clinical classification of PVR may help clinicians to better subclassify the clinical cases of PVR and allow an early diagnosis and treatment. A better identification of the high-risk cases is also necessary to target this group with prophylactic treatment before PVR is stablished. Furthermore, the adoption of a more homogenous surgical approach may be helpful for properly identifying novel adjunctive treatment options.

In the near future, larger, randomized clinical trials are required to better assess the possibility to adopt more effective adjunctive treatment options in combination with surgery for the management of PVR.
